# An Inquiry into the Physiological and Medicinal Properties of the Aconitum Napellus

**Published:** 1845-10

**Authors:** 


### Art. XVI.

*An Inquiry into the Physiological and Medicinal Properties of the Aconitum Napellus.* By A. Fleming, m.d. President of the Royal Medical Society of Edinburgh.—*London,* 1845. 8vo, pp. 160.

We were ageeably surprised by the contents of this book. Inaugural essays are for the most part at best the compilations of pains-taking able students; but this is an original examination by a careful and judicious observer of the effects of a powerful medicine, and is of importance not merely as illustrating the action of a single remedy of great power, but the action of a large class of medicines of which this may be taken as the type. Although we may neither admit that the Brunonian theory or the modern Italian improvement upon it explains the action of all medicines, yet we may accept as pretty true the leading principles of both, and yet conclude that neither explains the whole matter. We cannot believe with Brown that all medicines act as stimulants, but we agree with him that a large number of medicines produce indirect debility by having first occasioned over-excitement: and we freely agree with Rasori and Tommasini that there are remedies (contra-stimulants) which depress the vital powers at once without having first excited them. If this latter principle were doubted the facts related by Dr. Fleming of the action of aconite would supply convincing proofs of its truth; and as this medicine is a simple contra-stimulant, that is depressing the living power without previously exciting it, it is worth careful consideration. It gives us an opportunity of studying the symptoms of pure debility, which we need not say in practice is of great importance, as nothing is more difficult than to decide whether certain symptoms depend on mere weakness and require direct support, or are the result of indirect debility produced by over-excitement, and demand the opposite treatment.

When aconite is given to an animal it produces at first weakness of the limbs, and staggering and slightly quickened or else laborious breathing. The muscular paralysis increases, and there is loss of general sensibility; the animal lies down on his side with his limbs relaxed; he becomes blind; the breathing is slower and more imperfect, and, after a few spasmodic twitches, "death from asphyxia ensues." On examination immediately after death the heart is found beating strongly for some time, and the peristaltic action of the intestines continues. The voluntary muscles are less easily excited to contraction by mechanical irritation. The right side of the heart and the venous system are gorged with blood. The aconite produces no increased vascularity of the part to which it is applied. It acts most rapidly when introduced directly into the circulation; it produces no effect when applied to the skin; and acts with less energy when taken into the stomach than when introduced into a serous cavity or into the cellular tissue.

It acts in vegetables by depressing their vital powers. "A healthy plant, whose root is introduced into water impregnated with it, speedily begins to fade; its leaflets lose their freshness, hang over the edge of the glass, and soon shrivel and die."

When a small piece of the root is chewed, the flow of saliva is increased, and heat, tingling, followed by numbness of the lips and tongue, and a less free motion of the lips. "Its topical application is, as far as I have seen, unaccompanied either by pain, redness, or swelling, even when the physiological effects are developed to the fullest extent." When the ointment is applied to the conjunctiva, *contraction* of the pupil takes place and continues many hours: when applied to the temple or forehead, the pupil became, in two cases, dilated, attended with partial blindness. The reason of these opposite effects does not seem clear.

Its effects in medicinal doses are considered under four heads:

*First degree of operation.* Half an hour after a dose of 5 minims of the tincture, warmth is felt in the stomach, with slight nausea and oppression of breathing, followed in ten minutes by general warmth of the body, and numbness, tingling, and a sense of distension of the lips and tongue, continuing from one to three hours. Slight muscular weakness, with indisposition to exert either mind or body. In half an hour more, the pulse is weaker; and in another hour both pulse and breathing are slower, (say from 72 pulsations to 64, and respirations from 18 to 15 or 16.)

*Second degree.* After a second dose of 5 minims, two hours after the first, or after one dose of 10 minims, the symptoms are more severe and follow more quickly. Tingling down the arms, impaired sensibility of the surface, pulse 56, smaller and weaker, respirations 13, and labouring; great muscular debility, giddiness, and dizziness when standing, lethargy, unwillingness to be disturbed, and cold extremities. The symptoms last from three to five hours, and leave the patient languid.

*Third degree.* After another dose of 5 minims, two hours after the last, general warmth, with numbness and tingling spread rapidly over the body. Diminished sensibility of surface, countenance pale and anxious, headache, vertigo, dimness of vision, and muscular feebleness increased, voice weak, often a dread of approaching death, pulse occasionally falls to 40 or even 36, but more frequently rises to 70 or 80, and becomes small, weak, and irregular. Respiration irregular, short, and hurried, or deep and sighing. Surface moist and more cold. Sickness may come on. These symptoms do not subside for a day or two.

*Fourth degree.* If the medicine is continued, prostration increases, countenance sunken, sensation as if sinking from loss of blood, pulse smaller, weaker, and more irregular; breathing more imperfect; surface colder and covered with a clammy sweat. Consciousness usually remains. If the action is carried to a fatal extent, the individual becomes entirely blind, deaf, and speechless; pupils dilated, general muscular tremors, or even slight convulsions, pulse imperceptible, surface more cold, and after a few hurried gasps, death by syncope.

Of course, these symptoms do not occur in the same uniform manner in all cases.

The symptoms above enumerated indicate that aconite depresses the vital powers of the brain and nervous system of animal life, and of the heart and lungs. The languor and unwillingness to be disturbed by questions are symptoms of depression of the mental powers; the diminished sensibility of the skin, dimness and confusion of sight, or blindness and slight deafness, and the prostration of muscular strength, mark the weakness of the powers of animal life. The pulse at first diminished in strength, volume, and frequency, and at a later stage quicker and irregular, and respiration becoming in proportion slower and more laboured, show the debilitating action of aconite on the heart and lungs. The analogy between these symptoms and those produced by loss of blood strikingly confirms this view. The vertigo, tinnitus aurium, headache, and slight confusion of mind, are head symptoms common to both, and seem to be produced by a diminished flow of blood to the brain, as their severity is in proportion to the depression of the circulation, and they are aggravated by the erect posture.

Dr. Fleming draws many practical inferences from these premises. Such conclusions, before they are confirmed by actual experience, are not of great importance. It cannot, however, be doubted that aconite is a powerful antiphlogistic, and that its use is contraindicated in all states of the system depending on a debilitated state of the vital powers.

Death from aconite, as it has been observed in well-authenticated cases of poisoning in man where the fatal result was generally protracted for some hours, is produced by syncope. The symptoms have been described. In animals, where such a dose was given as produced death in a few seconds, it could not be attributed to syncope, as the heart's action continued after death; and not to asphyxia, as an animal may sustain complete suspension of breathing for at least three minutes. Whether we get nearer the truth by attributing death to "a powerfully sedative impression on the nervous system," as Dr. Fleming does, we will not attempt to decide. In animals where death is occasioned after an hour, it seems produced by asphyxia, as the heart after death is found beating, and there is engorgement of its right side. In cases of poisoning in man, the appearance of the symptoms depends on the nature of the preparation and state of the stomach. In one case where the tincture was taken, the symptoms appeared in a few minutes; in another, where the root was eaten, and there was food in the stomach, no unpleasant sensation was experienced for three quarters of an hour. Two drachms of the root, less than one drachm of the tincture, and four grains of the alcoholic extract have proved fatal. The treatment of poisoning (after vomiting) is obviously by stimulants. Brandy and hot water, and ammonia, with friction with warm cloths, especially along the spine.

*Therapeutic action of aconite.*

1. *Neuralgia.* Dr. Fleming has prepared a table of 40 cases: 10 of these have been published by others, and they are all permanent cures; of the 30 cases which fell under his own care, there were 17 permanent cures, and 13 where the relief was only temporary. In some of these the medicine was used internally, in others externally, sometimes both. Dr. Fleming suggests that if the neuralgia depends on inflammation either in the painful part of the nerve or further up in its course, or in sympathetic irritation, the internal use is more likely to be beneficial; if from local functional irritation, the topical application. Unless, however, there are well-marked indications of increased action of the vascular system, we should doubt the expediency of employing internally, in the first instance, so powerful a medicine. Should its external use fail, then (if there are no contra-indications) it might be given internally.

"I have met," says Dr. Fleming, "with several cases of neuralgia, in which the individuals had, for weeks or months, been in the habit of procuring sleep, and a temporary cessation of pain by opiate draughts, and who, on using the aconite, obtained permanent relief from the disease."

Dr. Fleming has tried it in 40 cases of *toothache,* by rubbing the gum with a few drops of the tincture, or by introducing a piece of cotton soaked with a drop or two into the carious tooth. In 7 of these it failed; in 6 it succeeded only for a short time; in the rest the relief was complete.

Of 15 cases of *headache,* 10 were relieved. Three of these were cases of nervous, four of plethoric, and three of rheumatic headache, that is headache of all kinds. We are all obliged often to treat this symptom empirically, and where it is troublesome, and there are no clear indications to the contrary, and attention to the stomach (the greatest source of headaches) has failed, there seems no reason why the external application may not be cautiously tried. A case is given in which the neuralgic pains attending aneurism of the abdominal aorta were somewhat relieved by its internal use.

2. *Rheumatism.* Störck first recommended aconite in acute rheumatism, and it has since been employed with success by many German, Swedish, and Swiss physicians.

Dr. Fleming exhibits in a tabular form 22 cases, 7 of which have been published by Lombard and Chandru, the others fell under his own care. The average period of cure was 5·6 days, the usual duration of treatment being about a fortnight or three weeks. In 3 cases a complete cure was effected in two days, in 1 in three days, and in 6 in four days. "The improvement following the administration of aconite is often very speedy, some alleviation of the pains being occasionally experienced in the course of an hour after the first dose has been taken, while there are few cases in which decided relief, with abatement of the redness, tension, and tenderness is not obtained in a few hours." Two only of the cases given were complicated with heart symptoms, and in both these had been observed before aconite was taken. Bouillaud, who bled largely, found one half of his patients had some heart complication in the first five days; and Dr. Macleod, who bled also, found symptoms of pericarditis in one fourth. "Thus," adds Dr. Fleming, "aconite not only effects a cure in a shorter period than any other mode of treatment, but appears to possess the great negative advantage of not increasing the liability to extension of the disease to the membranes of the heart. Indeed it seems rather to protect the patient from that dangerous complication." We fully agree with Dr. Fleming that "should *more extensive* trials confirm the conclusions drawn from the limited data here offered, its great superiority over the other ordinary modes of treatment will be undeniable." Dr. Fleming states that he has found it efficacious in the great majority of cases of chronic rheumatism, and that it possesses the great negative advantage of not weakening the strength, and impairing the constitution of the patient. Ten cases are given in a tabular form of lumbago, all of which were speedily cured by the internal use of the tincture combined with its external in some of them.

Mr. Liston has recommended aconite in erysipelas, and Dr. Fleming has employed it with marked benefit in several cases. In one case severe erysipelas of the leg of six days' standing was subdued in two days, and the pain in seven hours.

"The *tinctura aconiti* is prepared thus: Take of root of A. napellus carefully dried and finely powdered, sixteen ounces troy; rectified spirit, sixteen fluid ounces; macerate for four days; then pack into percolator; add rectified spirit, until twenty-four ounces of tincture are obtained." (p. 80.)

"I prefer," says Dr. Fleming, "the tincture for internal administration, from its greater uniformity of action.

"As an anodyne, antineuralgic, and calmative, five mimims ought to be given at first three times daily, to be increased daily to the extent of one minim each dose, until the physiological effects described under the second degree of operation have been produced. As an *antiphlogistic*, five minims ought to be given at first, and repeated in four hours, by which means the second degree of operation will, in all likelihood, have been induced. In order to sustain the sedative action, thus developed, two and a half minims are to be given every three or four hours, or less frequently, according to the effect produced. Where this mode of administration is adopted, it is *absolutely necessary* that the patient should be seen, and his pulse examined, before the exhibition of each dose. When this cannot be done, the remedy may be given in the manner pointed out in its use as an anodyne and calmative." (p. 81.)

We give Dr. Fleming's own words, but it is evident that the anodyne method is even a more powerful one than the antiphlogistic, as five minims three times a day will by many be interpreted to be a dose every four hours, and therefore the necessity is as great in this as in the other, of examining the pulse before the exhibition of a second dose. But we regard this dose as too high.

The high price of the alkaloid aconitina leads to a preparation of inferior quality or totally inert, being substituted in the shops. The tincture is an excellent substitute. "One or more drachms of it are to be rubbed over the affected part three times daily, the friction being continued at each time for a quarter of an hour, or until the topical effects of the drug are fully developed."

Several cases are given in an appendix, as well as experiments on animals.

In closing our analysis, we are merely doing justice to Dr. Fleming in expressing our high opinion of the merits of his essay. It is simple, clear, carefully considered, and judicious, and this is no slight praise to a young author writing on his favorite remedy, a dangerous theme even for a less sanguine time of life. Were we not thus satisfied, we should not have given further currency to the high recommendations of a remedy of such dangerous power: knowing how much more easy it is to go wrong than right, and that it is certain that it must do harm if administered without due consideration of the whole of the circumstances of the individual case, for which it is prescribed. We would therefore repeat our cautions, that it should be employed in no cases where there is great depression, or constitutional feebleness of the vital powers; that in nervous delicate women, especially of the upper classes who are great sufferers from neuralgia, it should not be thought of, unless externally where all simpler medicines had failed; but that when there is good vital power with increased vascular action attended with either neuralgic pains or the inflammation of the larger joints, it may be cautiously used, and where the effects of each dose cannot be watched, careful directions should be given that the medicine should not be long persevered in, and should be discontinued as soon as the patient complains of any feeling of sinking, or great cold, or depression. But we ourselves should be sorry to prescribe the remedy internally in any case which was not under our daily inspection.

Since the above notice was written, we have been much concerned to find that a gifted and estimable member of our own profession has died, as it would appear from taking aconite on the authority of this writer. Dr. Male of Birmingham, æt. 65, had complained of pains in the back and loins for two months; and not experiencing relief from ordinary means, he took tincture of aconite for four days, beginning with five-drop doses either twice or three times a day, and increasing the dose to six, eight, and ten drops, so that on the evening of the fourth day he had taken a dose of ten drops. On the morning of the fifth day he was seen by Mr. Russel. His extremities were cold, surface cold and clammy, pulse 130, feeble, cramps and pains in the legs, and spasmodic pains in the stomach; he expressed his conviction that he should die, and that the medicine was too powerful for him, but he also expressed his most earnest desire that he might recover, as his life was of the utmost importance to his children at this time. He gradually sank, and died on the morning of the seventh day without paralysis, and perfectly composed. The body was healthy; and the only morbid appearance was unusual fluidity of the blood. The verdict of the jury was "accidental death from an overdose of aconite taken medicinally by the deceased."

Mr. Russel was doubtful whether Dr. Male told him he had taken the aconite twice or three times a day. Had he taken it twice a day he would have taken 58 drops in four days, if three times a day 87 drops. From the result the latter is more probable, and also as Dr. Fleming recommends it to be taken three times a day. That the aconite killed him is most probable. The symptoms are precisely those described by Dr. Fleming—depression of all the vital powers; prostration, cold skin and extremities, rapid pulse, clear intellect, conviction of impending death. There were also some diarrhea, and spasmodic pains in the stomach from that acridity which produces heat and tingling in the mouth, but the fatal symptoms could in no way have depended on this comparatively slight local action. As there was no paralysis, loss of mental power, nor symptoms of asphyxia, death could neither be attributed to the effect of the poison on the brain and spinal cord, nor on the lungs. That aconite does not kill by immediately paralysing the heart is evident from experiments on animals, where the heart beats after death. May not, therefore, the general prostration of all the vital powers be explained by the poison destroying the life of the blood itself with which it must be mixed? And this view harmonizes with the fact that the only change discovered after death was increased fluidity of the blood.

"The treatment consisted in mild aperients with camphor and ammonia." To give aperients at all when there was such prostration, is very strange practice. The free use of very powerful stimulants, such as brandy, champaigne, wine, aided by electricity and friction, seems the rational means. They may have been employed, though no mention is made of them; and as two days elapsed between the last dose and death, there was plenty of time. The case unfortunately is a most instructive one. The responsibility which we feel in promulgating a favorable view of such a remedy is greatly increased by it. It is still more evident that aconite should never be given in slight cases or without the clearest indications that a powerful antiphlogistic is required, and that the age of the patient and his constitution warrant the belief in his vital energy. The case may be a most useful warning, and speak more powerfully than any reasoning as to the absolute necessity of caution in the use of aconite.

We are glad to be able to add from the pages of a contemporary, a brief notice of Dr. Male's life and character. He was a man deserving all consideration from his brethren.

"Dr. Male was the son of the late James Male, esq., of Belle Vue, near Birmingham. He received his preliminary education at Eton College, where he acquired some distinction in the classics and belles lettres. His medical studies were for the most part prosecuted in Edinburgh, from the university of which place he obtained his degree. He settled in Birmingham, as a physician, in 1802, and was shortly appointed one of the medical officers of the General Dispensary, which institution he continued to serve with much fidelity and usefulness for seven years. In 1805, the resignation of Dr. Carmichael caused a vacancy in the General Hospital, and Dr. Male was fortunate enough to become his successor. To this institution the subject of our biography was an honour and an ornament for thirty-six years; it is not too much to say of him, that for that lengthened period he was unvaryingly distinguished for the kindest sympathy and the profoundest skill, towards the several objects of his professional care. To his private patients, which were both numerous and of the highest respectability, he was endeared not only by the success of his treatment, but by the soundness and integrity of his principles. He was a man of most undeviating honour and rectitude, faithful to his friends, and of unyielding firmness in whatever he believed to involve matters of truth or of duty. He was a sincere upholder of professional dignity, courtesy, candour, and kindness. Perhaps the best proof of the estimation in which he was held by the profession of Birmingham, will be found in a remark made to the writer of this article by a fellow-physician, who is too just to pay an unmerited compliment, and too generous to withold a deserved one—the observation came with peculiar emphasis, for it was offered over a grave—'Dr. Male, sir, was a man to whom I invariably took my hat off.' That sentence, coming from a source whose uprightness none can better appreciate than ourselves, will convey a far higher tribute to the memory of the lamented individual, than any praise which it is in our power to offer.

"As an author, Dr. Male enjoyed a distinguished reputation. He was the father of English medical jurisprudence. The first work upon this subject in our language, was Dr. Samuel Farr's 'Elements of Medical Jurisprudence,' published in 1788. This, however, was little more than an abridgment of the 'Elementa' of Fazelius, and although medico-legal questions were subsequently discussed in the writings of Mead, Munro, Denman, Percival, and John Hunter, and Dr. William Hunter published an able essay 'On the Uncertainty of the Signs of Murder in the case of Bastard Children,' yet the first *original* work in English on medical jurisprudence, was Dr. Male's 'Epitome of Juridical or Forensic Medicine.' This appeared in 1816, was dedicated to Sir Samuel Romilly, and consisted of 200 pages. It displayed an unusual amount of shrewd observation, clear reasoning, and comprehensive research. It shortly passed through a second edition, and was reprinted by Dr. Cooper in America, in 1819, along with the treatises of Farr and Haslam. Although various impediments, professional and other, prevented Dr. Male from bringing it in later years up to later science, his work is still quoted as being upon certain subjects amongst the most exact and accurate of any extant.

"It is singular that Birmingham should be able to boast, in the person of the late Dr. Male, of the first original writer on English medical jurisprudence, and in the person of the late Dr. Darwall, of the most ample annotator of the greatest work upon the subject in our language and out of our country—viz., the stupendous work of Beck in America; and the coincidence is not more remarkable than mournful, that the distinguished individual whose memoir we now close, should have been one of our first writers on indigenous poisons, and finally a victim of the deadliest of them." (Medical Times, August 9, 1845.)

---

